# Geospatial mapping of distribution grid with machine learning and publicly-accessible multi-modal data

**DOI:** 10.1038/s41467-023-39647-3

**Published:** 2023-08-17

**Authors:** Zhecheng Wang, Arun Majumdar, Ram Rajagopal

**Affiliations:** 1 Department of Civil & Environmental Engineering, Stanford University, Stanford, CA 94305 USA; 2 Department of Electrical Engineering, Stanford University, Stanford, CA 94305 USA; 3 Department of Mechanical Engineering, Stanford University, Stanford, CA 94305 USA; 4 Department of Energy Science & Engineering, Stanford University, Stanford, CA 94305 USA

**Keywords:** Energy grids and networks, Geography, Energy access, Developing world, Computer science

## Abstract

Detailed and location-aware distribution grid information is a prerequisite for various power system applications such as renewable energy integration, wildfire risk assessment, and infrastructure planning. However, a generalizable and scalable approach to obtain such information is still lacking. In this work, we develop a machine-learning-based framework to map both overhead and underground distribution grids using widely-available multi-modal data including street view images, road networks, and building maps. Benchmarked against the utility-owned distribution grid map in California, our framework achieves > 80% precision and recall on average in the geospatial mapping of grids. The framework developed with the California data can be transferred to Sub-Saharan Africa and maintain the same level of precision without fine-tuning, demonstrating its generalizability. Furthermore, our framework achieves a R^2^ of 0.63 in measuring the fraction of underground power lines at the aggregate level for estimating grid exposure to wildfires. We offer the framework as an open tool for mapping and analyzing distribution grids solely based on publicly-accessible data to support the construction and maintenance of reliable and clean energy systems around the world.

## Abstract (web summary)

Granular geospatial information of distribution grids is needed for various power system applications. Here the authors develop a machine-learning-based model which can accurately map distribution grids in both the U.S. and Sub-Saharan Africa.

## Introduction

Detailed and accurate mapping of power grids is essential for power system planning, operation, and risk management around the world. In Sub-Saharan Africa (SSA), the proportion of the population without access to electricity was still over 50% in 2020^1^. Despite increased connections, SSA countries experience on average 50 to 4600 hours of power outage out of the 8760 hours in a year due to limited capacities and infrastructure failures^2,3^. Expanding and upgrading electricity transmission and distribution infrastructures requires detailed information on their current locations, connections, and status.

Meanwhile, the deployment of distributed energy resources (DERs), such as solar photovoltaics (PV), is growing rapidly worldwide. Distributed generation is projected to account for 10% of the total global power generation by 2030^4^. The deep penetration of DERs into power grids poses significant challenges to grid stability due to the bidirectional power flow created by DERs. To monitor DERs and integrate them into power systems, detailed information on grids, especially of distribution grids, is a prerequisite.

Unfortunately, unlike transmission grids of which the connections and status are usually available to system operators and can be regularly measured^5,6^, information on distribution grids is often incomplete, coarse-grained, or even unavailable, especially in developing countries^7^. Even in developed countries, although utility companies may keep the information of their own distribution grids, such data are usually not publicly-available or organized in a standardized format^8^, which forms sequestered data silos and prevents researchers and policymakers from analyzing grid status and developing investment plans across different utility territories. OpenStreetMap maintains a geospatial data collection of power lines by utilizing crowdsourcing methods, yet it is far from complete, and most of the data in this collection are for transmission lines^9^.

Previous graph-based approaches for distribution grid topology estimation rely on the availability of measurement data collected at the nodes (i.e., buses) of distribution grids, e.g., time-series observations from smart meters^6,10–14^. Despite the rapid deployment of smart meters in developed economies, many countries in Africa and Latin America still have a low deployment rate of smart meters^15^, and these meter data, if present, are privately owned by different utility companies. Due to such limitations, the graph-based approaches are useful in the operational topology identification of partially-known grids with measurements at nodes rather than mapping real-world physical distribution grids completely from scratch without any prior knowledge of nodes or edges.

With the rapid development of machine learning and computer vision in recent decades^16^, there are attempts to automatically detect energy transmission and distribution infrastructures utilizing publicly-available imagery data. Arderne et al.^8^ developed a predictive model for mapping medium-voltage distribution grids in developing countries by connecting the electrified settlements detected in night-time light imagery. However, the spatial granularity of the grid map generated by such an approach is limited by the resolution of night-time light imagery which is only at the level of kilometer or hundreds of meters^17^. Schmidt et al.^18^ developed a machine-learning model to extract voltage rating information of transmission lines from aerial images. Gomes et al.^19^ and Huang et al.^20^ used deep learning to localize utility poles and predict line connections from remote sensing images, but the applicability of such methods on distribution grids is subject to varying image resolutions and qualities of remote sensing images across different places, as utility poles and distribution lines, compared with transmission infrastructure, are barely visible from low-resolution or noisy remote sensing images. Deep learning has also been used to either localize or analyze utility poles in street view images^21–24^, but not for identifying power line connections to construct a full distribution grid map. Furthermore, methods based solely on remote sensing or street view images are not able to detect and map underground power lines.

In this work, we propose a general machine-learning-based framework to construct distribution grid maps by combining multi-modal widely-available data including street view images, road networks, and building locations. Convolutional Neural Networks (CNN)—trained with only image-level class labels in a weakly-supervised manner—are used to detect and estimate the orientations of utility poles and power lines from upward street view images. Leveraging power line detection results and road networks as features, a link prediction model is used to predict the line connections between utility poles. We further predict the geospatial map of underground grids on top of the predicted overhead grid map by incorporating the information of road networks and building locations. The entire framework is developed and evaluated using the data in California with utility-owned distribution grid maps as a benchmark. The framework is also transferred to the test areas in three cities in Sub-Saharan Africa and shows reasonable performance without re-training or fine-tuning. We offer our framework as a general tool to map distribution grids, which can be further combined with other geospatial data, such as tree locations, DER inventory, and weather data, to facilitate various power systems applications such as electricity access expansion, vegetation management, DER integration, and risk assessment.

## Results

### Overall framework

**Fig. 1:**
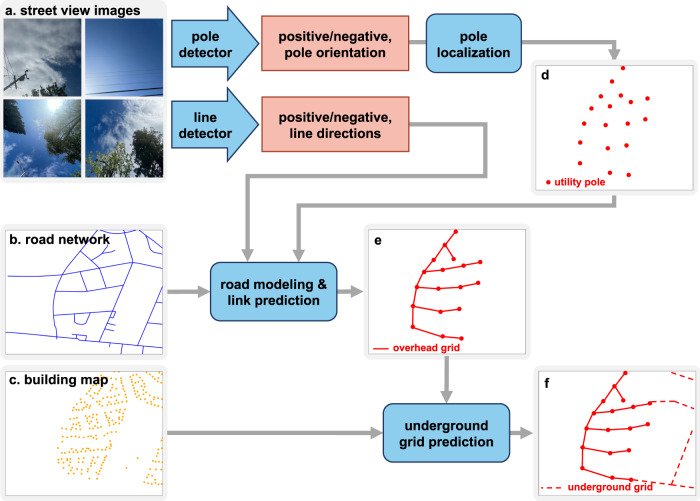
The overall framework of distribution grid mapping. Blue arrows and blocks represent individual modules, while light red blocks represent output variables. **a**–**c** Inputs include: **a** street view images; **b** road network; **c** a map of building locations. Street view images are processed by two weakly-supervised Convolutional Neural Networks (CNNs), a pole detector and a line detector, to estimate pole orientations and line directions, respectively. **d** Pole orientations are used to localize poles. **e** Pole and line information are integrated with the road network to predict power line connections between poles. **f** Building locations and the predicted overhead grid map are integrated to predict the underground grid map.

**Fig. 2:**
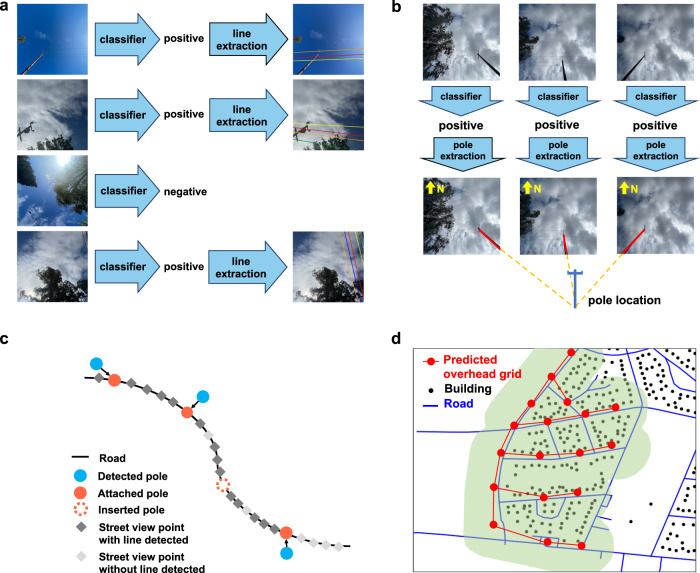
Illustration of different modules. **a** Power line detector. It first decides whether there is any line in the image (positive) or not (negative), and if there is, then estimates its direction. **b** Utility pole detector. It first decides whether there is any pole in the image (positive) or not (negative), and if there is, then estimates the pole orientation. Rays of pole orientations estimated from multiple images are intersected to obtain pole locations. **c** Road modeling. Street view points and the detected poles are attached to nearby roads. To reduce the number of poles missed by the pole detector, an additional pole is inserted between a pair of poles if the distance between them is greater than a distance threshold *D*_insert_. **d** The heuristic approach for underground grid mapping, which is based on the assumption that all buildings have access to the grid. Lines of the predicted overhead grid are dilated to cover nearby buildings. Then a modified Dijkstra’s algorithm is used to connect the uncovered buildings to the grid by incorporating the prior knowledge of the geolocation preference of underground line constructions. The paths generated for connecting these buildings are used as the prediction of the underground grid connections.

The schematics of the overall framework is shown in Fig. 1. Street view images, captured at different geolocations, are used to localize utility poles and estimate the directions of power lines. Specifically, a CNN-based power line detector takes street view images as inputs and outputs classification results indicating whether an image contains power lines and, if containing lines, the estimated line directions (Fig. 2a). Similarly, a CNN-based utility pole detector takes street view images as inputs and outputs classification results indicating whether an image contains poles or not and, if containing poles, the predicted pole orientations. Pole orientations estimated in multiple images are combined together to localize poles by intersecting the rays that represent pole orientations (Fig. 2b). Both line detector and pole detector are trained in a weakly-supervised manner—only image-level class labels are provided as supervision, while pole orientations or line directions are not due to the expensive cost of obtaining their ground truth annotations (see details in Methods).

Unlike previous works^21–23^ that have used the horizontal perspective of street views, we instead use upward street views as there are much fewer irrelevant objects and, furthermore, the geometric relationships are simpler to capture in the upward view. By setting the street view orientation to be uniformly facing north, we can directly estimate line directions and pole orientations relative to the street view point (see details in Methods).

Both the line detector and the pole detector are trained, validated, and tested on a dataset containing 10,000 upward street view images randomly sampled from the San Francisco Bay Area (see details about the dataset in Methods). We use precision (ratio of correct decisions among all positive decisions) and recall (ratio of correctly-identified samples among all positive samples) to measure the image-level classification performance. For line detection, the model achieves a precision of 0.982 and a recall of 0.937 on the test set. For pole detection, the model achieves a precision of 0.982 and a recall of 0.850 on the test set.

Pole and line information extracted from street view images are then integrated with the road network to predict line connections between predicted poles. We first establish the geospatial relationship between poles, street view points, and roads. Specifically, for each predicted pole or street view point, if there is a nearby road, it will be attached to that road (Fig. 2c). A machine-learning-based link prediction model is used to predict whether there is a line connection between a pair of poles by leveraging features such as whether there is any line detected in street view images between the two poles, and whether the two poles are next to each other along the road (see details of the used features and feature selection in Methods). We, therefore, obtain a geospatial graph representing the predicted overhead grid, with predicted poles as nodes and predicted line connections as edges.

The prediction of underground grid map is based on the assumption that all buildings are connected to the grid—if a building is not connected to the nearby overhead grid, then it should be connected to the nearby underground grid. This assumption generally holds in countries with a 100% electrification rate for buildings, but does not hold in areas with buildings unelectrified or self-powered. In the U.S., for example, the number of people living off-the-grid is less than 0.1% of the total population^25^. Note that microgrids and distributed generation can make buildings detached from utility grids, but buildings still need to be connected to utility grids either in normal operations or before they go isolated. Under this assumption, we overlay the predicted overhead grid map with the building map to identify the buildings which cannot be reached by the predicted overhead grid within a certain distance (Fig. 2d). As a heuristic approach, we predict the geospatial map of the underground grid by running a modified Dijkstra’s algorithm^8,26,27^ to find the most efficient paths to connect these unconnected buildings by incorporating the prior knowledge of the geolocation preference of underground line constructions (e.g., roads are preferable. See details in Methods). While the predicted overhead grid is represented as a geospatial graph, the predicted underground grid cannot be explicitly represented as a set of nodes and edges hence presented in the raster format.

### Performance in the California test areas

Based on a distribution grid map of the largest utility company in California^28^, Pacific Gas and Electric Company (PG&E), we curate a dataset covering the distribution grids in 6 different areas in California (San Carlos, Newark, Santa Cruz, Yuba City, Pacific Grove, and Salinas. See details about the dataset in Methods). The distribution grid map in San Carlos is used as a development set for training and validating the link prediction model, while the remaining 5 areas in California are used as the test set to evaluate the pole localization performance, link prediction performance, and overall grid mapping performance.

The performance of pole localization is evaluated with two metrics: (1) precision, defined as the fraction of detected poles that are within a distance *D*_matching_ of a ground truth pole, and (2) recall, defined as the fraction of ground truth poles that can be detected within a distance *D*_matching_ (see details of the metrics in Methods). Table 1a shows the pole localization performance in the California test areas with *D*_matching_ = 25m. For the 5 test areas, over 80% of the ground truth poles can be detected within 25m (“recall”) except in Yuba City (78%), and over 80% of the predicted poles have a nearby ground truth pole within 25m (“precision”) except in Pacific Grove (77%). Furthermore, our framework can localize the poles that have not been documented in the PG&E distribution grid map (Table 1a), and these newly-detected poles can serve as supplements to the utility-owned data. We verify these supplemented poles by the visual inspection of nearby street views and remote sensing images. By taking them into consideration, the overall F1 score (harmonic mean of precision and recall) of pole localization in the California test areas ranges from 0.83 to 0.91.

**Table 1:** Pole localization performance

Test area	Precision	Recall	F1 score	# supplemented poles	Precision (after supplement)	Recall (after supplement)	F1 score (after supplement)
**a. Test areas in California**							
San Carlos, CA, U.S.A. (development set)	0.836	0.800	0.818	146	0.895	0.807	0.849
Newark, CA, U.S.A.	0.845	0.811	0.828	95	0.916	0.820	0.865
Santa Cruz, CA, U.S.A.	0.850	0.818	0.834	47	0.890	0.824	0.856
Yuba City, CA, U.S.A.	0.880	0.776	0.825	9	0.887	0.778	0.829
Pacific Grove, CA, U.S.A.	0.773	0.832	0.801	123	0.848	0.839	0.844
Salinas, CA, U.S.A.	0.816	0.943	0.875	73	0.888	0.939	0.913
Average (except San Carlos)	0.833	0.836	0.833	69.4	0.886	0.840	0.861
**b. Test areas in Sub-Saharan Africa**							
Ntinda, Kampala, Uganda	0.799	0.707	0.750	-	-	-	-
Kololo, Kampala, Uganda	0.853	0.717	0.779	-	-	-	-
Highridge, Nairobi, Kenya	0.895	0.647	0.751	-	-	-	-
Ngara, Nairobi, Kenya	0.887	0.584	0.704	-	-	-	-
Ikeja, Lagos, Nigeria	0.953	0.636	0.763	-	-	-	-
Average	0.877	0.658	0.749	-	-	-	-

Distance threshold *D*_matching_, which is the maximum allowable distance for matching a predicted pole with a ground truth pole, is set to be 25m. F1 score is the harmonic mean of precision and recall.

**Fig. 3:**
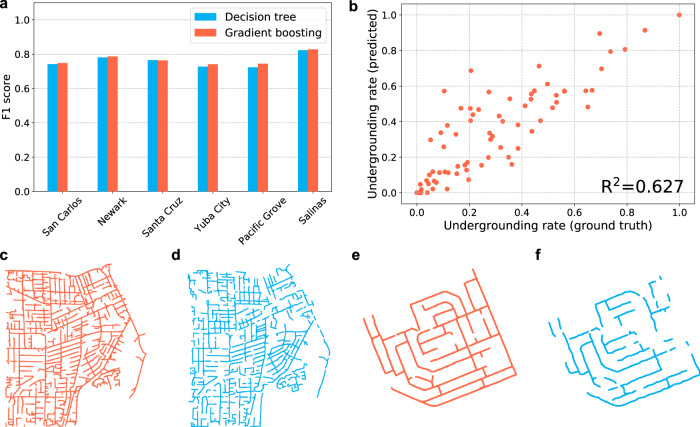
Model performances and distribution grid map visualization. Predicted and ground truth grid maps are visualized in the raster format. **a** Comparison between two link prediction models, decision tree and gradient boosting, across the 5 test areas in California. The F1 score is the harmonic mean of precision and recall. **b** The scatterplot of the predicted values of undergrounding rates versus ground truth values at the census block group level for the test areas in California. Undergrounding rate is defined as the fraction of underground lines in terms of length in a block group. *R*^2^ is the coefficient of determination. **c** Ground truth grid map for the test area in Yuba City, CA, U.S.A. **d** Predicted grid map for the test area in Yuba City, CA, U.S.A. **e** Ground truth grid map for the test area in Ikeja, Lagos, Nigeria. **f** Predicted grid map for the test area in Ikeja, Lagos, Nigeria.

By comparing the predicted line connections with the ground truth ones, we evaluate the performance of link prediction models using precision (fraction of predicted lines that are correct) and recall (fraction of ground truth lines that can be detected), as well as their harmonic mean, F1 score (see details of the metrics in Methods). Figure 3a compares two link prediction models—decision tree and gradient boosting—in terms of the F1 scores across the 5 test areas, and it shows that gradient boosting performs slightly better than decision tree. For the gradient boosting model, the precision of link prediction ranges from 0.71 to 0.83 across the 5 test areas, while the recall ranges from 0.67 to 0.89 (Table 2a). Similar to the localization of supplemented poles, our framework can identify additional line connections that are not documented in the utility-owned grid map. After considering these unrecorded connections as supplements, the model can achieve a F1 score from 0.75 to 0.87 across the 5 test areas, with the highest in Salinas (0.87) and the lowest in Yuba City (0.75).

**Table 2:** Link prediction performance

Test area	Precision	Recall	F1 score	# supplemented edges	Precision (after supplement)	Recall (after supplement)	F1 score (after supplement)
**a. Test areas in California**							
San Carlos, CA, U.S.A. (development set)	0.787	0.713	0.748	161	0.853	0.726	0.784
Newark, CA, U.S.A.	0.816	0.760	0.787	113	0.899	0.773	0.832
Santa Cruz, CA, U.S.A.	0.809	0.723	0.763	48	0.851	0.730	0.786
Yuba City, CA, U.S.A.	0.828	0.671	0.741	9	0.836	0.673	0.746
Pacific Grove, CA, U.S.A.	0.709	0.783	0.744	132	0.780	0.793	0.786
Salinas, CA, U.S.A.	0.774	0.891	0.828	85	0.856	0.889	0.872
Average (except San Carlos)	0.787	0.766	0.773	77.4	0.844	0.772	0.804
**b. Test areas in Sub-Saharan Africa**							
Ntinda, Kampala, Uganda	0.801	0.664	0.726	-	-	-	-
Kololo, Kampala, Uganda	0.826	0.639	0.721	-	-	-	-
Highridge, Nairobi, Kenya	0.879	0.701	0.780	-	-	-	-
Ngara, Nairobi, Kenya	0.793	0.510	0.621	-	-	-	-
Ikeja, Lagos, Nigeria	0.908	0.637	0.749	-	-	-	-
Average	0.841	0.630	0.719	-	-	-	-

**a** Performance in the California test areas. **b** Performance in the Sub-Saharan Africa test areas.

Results with gradient boosting model used for link prediction are shown. Edges between false negative poles (poles that are not detected) are counted as false negative edges, and edges between false positive poles (wrongly-detected poles) are counted as false positive edges. F1 score is the harmonic mean of precision and recall.

To evaluate the overall grid mapping performance covering both overhead and underground grids, we define precision as the fraction of predicted distribution grid located within a distance *R*_eval_ of ground truth grid, and define recall as the fraction of ground truth distribution grid that can be detected within a distance *R*_eval_ (see details of the metrics in Methods). The results across the California test areas, obtained with the gradient boosting as the link prediction model, are shown in Table 3a (*R*_eval_ = 20m). As is shown, across the 5 test areas in California, 76–93% of the ground truth distribution grid can be detected within 20m (“recall”). For 75–92% of the predicted distribution grid, ground truth distribution grids can be found within 20m (“precision”). Figure 3c and d show the visualization of the ground truth and the predicted distribution grid maps in the test area of Yuba City, respectively.

**Table 3:** Overall grid mapping performance

Test area	Precision	Recall	F1 score
**a. Test areas in California**			
San Carlos, CA, U.S.A. (development set)	0.857	0.797	0.826
Newark, CA, U.S.A.	0.850	0.805	0.827
Santa Cruz, CA, U.S.A.	0.751	0.766	0.758
Yuba City, CA, U.S.A.	0.875	0.762	0.815
Pacific Grove, CA, U.S.A.	0.840	0.871	0.856
Salinas, CA, U.S.A.	0.924	0.926	0.925
Average (except San Carlos)	0.848	0.826	0.836
**b. Test areas in Sub-Saharan Africa**			
Ntinda, Kampala, Uganda	0.920	0.782	0.846
Kololo, Kampala, Uganda	0.962	0.782	0.863
Highridge, Nairobi, Kenya	0.971	0.802	0.878
Ngara, Nairobi, Kenya	0.982	0.655	0.786
Ikeja, Lagos, Nigeria	0.988	0.756	0.857
Average	0.965	0.755	0.846

**a** Performance in the California test areas. Supplemented poles and line connections are considered, and the results are for the combination of underground and overhead parts of the grid. **b** Performance in the Sub-Saharan Africa (SSA) test areas. Only the overhead part of the grid is considered, as the benchmark for underground grid map is not available in SSA.

The performance is evaluated on a raster map using path dilation with a dilation radius *R*_eval_ = 20m (see details in Methods). F1 score is the harmonic mean of precision and recall. Gradient boosting model is used for link prediction.

For the California test areas, incorrect underground grid mapping is a major source of error contributing to 82% of false positive mapping and 71% of false negative mapping due to the heuristic nature of the underground grid mapping approach. A potential mitigation approach is to incorporate other prior knowledge besides road maps (e.g., topographic map and land cover map) to reflect additional geolocation preferences of underground lines for the modified Dijkstra’s algorithm. See detailed analysis of sources of error in Supplementary Note 2.

### Performance in the Sub-Saharan Africa test areas

To evaluate the generalizability of the grid mapping framework, especially in developing countries where the infrastructure data are scarce, we transfer the framework developed using the data in California to 5 manually-curated test areas in three countries in Sub-Saharan Africa (SSA): Uganda, Kenya, and Nigeria (see details about the dataset in Methods). Among them, Uganda is considered as a low-income country, while Kenya and Nigeria are considered as lower-middle income countries according to the World Bank country classification^29^. We evaluate the model performance in these test areas with the same metrics as in California. The line detector, the pole detector, and the link prediction model all remain the same without re-training or finetuning. All hyperparameters are also the same as those used in California except that the decision threshold to classify an image as positive is changed from 0.5 to 0.2 for the pole detector. Such a change is based on the observation that the utility poles in Sub-Saharan Africa are generally shorter than those in the U.S. which can make them more difficult to identify in upward street view images. Figure 3e and f show the ground truth and the predicted distribution grid maps for the test area in Ikeja, Lagos, Nigeria. Note that we do not predict the underground grid map for the SSA test areas since the assumption for underground grid mapping—all buildings are connected to the grid—does not necessarily hold in SSA, and the reference underground grid maps in SSA are not available for model evaluation.

Table 1b shows the pole localization performance. While precisions of pole localization across the SSA test areas are generally higher than 0.8, the recall drops from an average of 0.84 in the California test areas to an average of 0.66 in SSA, which can be largely attributed to the missing detection of poles in images (contributing to 71% of the false negative pole localization. See Supplementary Note 2 for details). Such missing detection is partially due to the difference in the appearance of utility poles between the U.S. and SSA. Moreover, some utility poles in SSA are comparatively short, making them out of sight in upward street view images if they are not close enough to the locations where street view images were captured (contributing to 24% of the false negative pole localization). Missing pole detection further affects the link prediction performance—the recall drops from an average of 0.77 in the California test areas to an average of 0.63 in SSA (Table 2b). A potential mitigation approach is to augment the field of view (FoV) of upward images by leveraging the panoramic street views (see a discussion on sources of error and mitigation approaches in Supplementary Note 2). Nevertheless, the framework achieves a precision from 0.92 to 0.99 and a recall from 0.66 to 0.80 in overall grid mapping (Table 3b), indicating that our framework, trained with the data in the U.S., can maintain a high correct rate and a reasonable detection rate of mapping when transferred to SSA without re-training or fine-tuning.

### Estimate the fraction of underground power lines

The grid map predicted by our framework can be used for various power systems applications such as grid maintenance and risk assessment. For example, exposed overhead power lines are not only vulnerable to wildfire-caused damages but can also ignite wildfires by sparks and intruding vegetation. For example, the Dixie Fire in 2021—the largest non-complex wildfire in California’s history that burned over 960,000 acres—was caused by distribution lines contacting a tree^30,31^. Burying power lines is an effective but costly approach to mitigate such a coupled risk of wildfires and power grids^32^. Although some utilities may maintain the information of line-burying status of their own distribution grids, such data is privately owned by various stakeholders as data silos and not publicly available. Obtaining the map of line burying status with high spatial granularity in an automated, scalable, and non-intrusive way can facilitate the assessment of grid exposure to wildfire risks across different utility territories, identification of disparities in infrastructure vulnerability, and the development of wildfire risk mitigation policies and infrastructure investment plans.

Leveraging the geospatial map of overhead and underground grids predicted by our framework, we can estimate the total length of underground and overhead lines as well as their ratio in a given area. We evaluate the accuracy of our grid mapping method in estimating line-burying status by comparing the predicted and ground truth values of the undergrounding rate—defined as the fraction of underground lines in terms of length—in each census block group which is a small geographical unit defined as by the U.S. Census Bureau that typically contains 600 to 3000 people^33^. We use the 73 block groups across the 5 test areas in California as the test set (excluding block groups in the development set of San Carlos), on which our model can achieve a prediction *R*^2^ of 0.627. The scatterplot of the predicted values versus ground truth values is shown in Fig. 3b. Considering that the mapping of underground lines is purely based on the publicly-available building and road data, such a performance is reasonable, demonstrating the effectiveness of our framework in revealing heterogeneous line-burying status at a granular geographic aggregation level. The derived information of the aggregate-level line-burying status can be further combined with the geospatial distribution of wildfire risks and vegetations to assess the regional vulnerability of distribution grids to wildfires and to prioritize the areas for infrastructure investment towards a wildfire-resilient energy system.

## Discussion

Due to the small visual targets, complexity of graph structures, and combination of both overhead and underground power lines, mapping distribution grids is a challenging task requiring the integration of different modalities of data. In this work, we develop a machine-learning-based framework combining widely-available multi-modal data including street view images, road networks, and building maps to predict the geospatial map of distribution grids. Its performance is extensively evaluated across the test areas in both California in the U.S. and Sub-Saharan Africa (SSA), which shows the generalizability of our framework. The image-level performance is also evaluated in the test areas in six additional cities from Africa, Asia, and Latin America (see Supplementary Note 4 and Supplementary Table 2). Sources of error for both the California and SSA test areas and the potential mitigation approaches are discussed in Supplementary Note 2, Supplementary Figures 4 and 5.

Our proposed framework has three advantages. First, compared with measurement-based methods, our framework is not restricted by the availability of measurement data or prior knowledge of node locations. The input data is readily-available—street view images are collected by both technology companies such as Google, Microsoft, and Baidu, as well as volunteer contributors^34^, and their coverage is rapidly expanding around the world including underdeveloped countries; road networks and building locations are widely available from OpenStreetMap^9^. Second, our framework is not subject to the heterogeneous resolution and quality of remote sensing imagery. It is also weakly-supervised—only image-level class labels are needed for training the models to estimate power line directions and localize poles from images, eliminating the need for labor-intensive object annotations^20,21^. Third, unlike previous methods that are purely based on images, our framework can predict the geospatial map of underground grids on top of the predicted overhead grid map using non-imagery data.

We note three limitations. First, street view images are comparatively scarce in some developing countries at present. However, with the rapidly increasing coverage of street view imagery around the world including rural areas in developing countries, as well as the participation of volunteer street view contributors^34^, such an issue is expected to be mitigated over time. Grid maps can be expanded or updated by our framework along with the arrival of new imagery data. There is also a potential to incorporate local geospatial patterns of utility pole placement into the framework to reduce the number of images needed for pole localization (see detailed discussion in Supplementary Note 5). Moreover, as our framework has a module that integrates road and building information for the heuristic mapping of underground grids, for areas without street view images, the framework can still work as a heuristic approach to estimate the entire grid—not just the underground part—by utilizing road and building information. The assumption (e.g., building connectivity assumption) underlying this heuristic approach can also be empirically examined in future work.

Second, utility poles that are short or far away from street view points may not be captured in upward street views, which can contribute to the false negative detections of utility poles (see Supplementary Note 2). Such a limitation can be alleviated by augmenting the field of view (FoV) of street view images with full panoramas which are commonly captured in street view photography. Also, differences between the appearance of utility poles across different countries can lead to false negative detections when the model is transferred to a new country. To tackle this, fine-tuning or few-shot learning can be leveraged to adapt the model to a new region with a small amount of additional training samples, which deserves future exploration.

Third, the electrical parameters of power lines and the exact operational topology of distribution grids cannot be captured by our current framework which primarily focuses on geospatial mapping. However, machine learning has the potential to be applied on street view images to further identify transformers and switches, and to estimate fine-grained characteristics (e.g., voltage level) of power lines by leveraging rich visual features in street views (e.g., pole/line height, the quantity of insulators) and by incorporating domain knowledge into machine learning models, which is also part of our future work.

Based on the geospatial grid maps generated by our framework, learning-based models can also be developed to examine whether a power line is severely intruded by tree branches, and whether a utility pole is vulnerable to extreme weather conditions (e.g., leaning^23^) which can potentially affect grid safety and reliability. Other geospatial data, such as the spatial distribution of wildfire risks, vegetation maps, and predictive maps of extreme events such as storms and hurricanes, can also be integrated and superimposed with the generated grid maps to estimate the exposure of grid infrastructure to natural hazards and extreme events across different places amidst the growing threat of climate change. Further, such a map overlay can be used to support grid infrastructure planning and management, such as the prioritization of system hardening for risky poles and power lines, vegetation management, and the identification of buildings that likely lack access to grids for evaluating electricity access expansion projects in SSA. Additionally, the proposed approach or its variant has the potential to be applied for mapping and estimating the status of other energy or utility assets and infrastructures such as telecommunication cables, electrical transformers, and street lights, which deserves future exploration.

## Methods

### Datasets

We construct a street view image dataset to train the line detector and pole detector. The dataset contains 10,000 upward street view images which are randomly sampled from the San Francisco Bay Area. Each image is retrieved with Google Street View Static API^35^ (see more details in Supplementary Note 3) and manually annotated with two binary labels indicating whether it contains lines and whether it contains poles, respectively. There are 3,204 images containing line(s) among which 1,786 images contain pole(s). 14% of the images containing poles/lines have nearby trees or buildings overlapped with poles/lines (i.e., not backgrounded by clear sky). The dataset is split into training, validation, and test sets following the 85%-7.5%-7.5% ratio.

To develop the link prediction model and evaluate the grid mapping performance, we collect and process distribution grid maps in 11 different areas and treat them as ground truth distribution grid maps. 6 of them are from cities in California in the U.S., including San Carlos, Newark, Santa Cruz, Yuba City, Pacific Grove, and Salinas. For these 6 areas, we obtain the geospatial maps of distribution grids from the Integration Capacity Analysis (ICA) map^28^ of Pacific Gas and Electric Company (PG&E), and then manually distinguish between overhead and underground power lines by examining street view and remote sensing images. Overhead grids can be represented as geospatial graphs containing nodes corresponding to utility poles and edges corresponding to power lines.

Other 5 test areas are from three cities in SSA, including two areas in Kampala, Uganda, two areas in Nairobi, Kenya, and one area in Lagos, Nigeria. These test areas cover both taller utility poles with complicated structures as well as shorter and simpler poles (e.g., without crossarms) for representativeness (see examples in Supplementary Fig. 6). The World Bank maintains a geospatial dataset of transmission and distribution grids in Africa^36^, but it only covers a few cities and most of the data in this dataset are for transmission lines. We correct errors in this dataset and identify additional overhead distribution lines by manually checking street view images and remote sensing images, and eventually construct the distribution grid maps for the 5 test areas in SSA that serve as the ground truth for model evaluation.

Road maps, which can be represented as geospatial graphs with nodes and edges, are obtained from OpenStreetMap^9^ for the test areas in both California and SSA. The building map contains a set of geo-coordinates for buildings. For the test areas in California, the building data are obtained from the Microsoft US Building Footprints dataset^37^, while for the test areas in SSA they are obtained from OpenStreetMap^9^.

### Weakly-supervised line extraction and pole localization

Each upward street view image is processed by two Convolutional Neural Networks (CNNs)—a power line detector and a utility pole detector. The line detector classifies an image into either positive (contain lines) or negative category (no line found), and then extracts the line directions for positive images (Fig. 2a). Similarly, the pole detector classifies the image and then estimates the pole orientations (Fig. 2b). Both CNNs use Inception-v3^38^ as the backbone architecture (i.e., main branch) with a segmentation branch added to an intermediate layer of the main branch for generating class activate maps (CAMs) (see Supplementary Figure 1 for overall model architecture). A CAM is a heat map highlighting the activated area of target objects^39^ by deriving the weighted sum of feature maps. We choose an intermediate layer at the middle point of the main branch to add the segmentation branch and generate CAM, as the feature maps learned at upstream layers are noisy but of high resolution, while the feature maps learned at downstream layers are specific but of low resolution. Using the feature maps at the middle of the network can balance such a trade-off.

In our work, we use CAMs to extract the locations and directions of power lines or utility poles in an image. A significant advantage of CAM is that it can be obtained in weakly-supervised manner—only image-wise labels indicating positive or negative are needed to train the model to gain the capability of extracting target objects. Such a method eliminates the need for manually-labeled line or pole annotations in training images which are highly labor-intensive to obtain. Supplementary Figs. 2 and 3 show the examples of CAMs for line extraction and pole detection, respectively.

For training line detector or pole detector, we first train the main branch of the model for classification, and then freeze the main branch when training the segmentation branch to develop the ability to generate CAM. In the training phase, the segmentation branch is trained with only image-level binary labels as supervisory signals for classification (i.e., weakly supervised), but it is used to generate CAMs in the inference phase. We freeze the main branch when training the segmentation branch, as we want to keep the classification ability of the main branch unchanged and rely on it instead of the segmentation branch for image-level classification in the inference phase (see illustration of the training and inference phases in Supplementary Fig. 1).

In the training phase of both the main branch and the segmentation branch, each input image is rotated with an angle randomly selected among 0°, 90°, 180°, and 270° and also randomly flipped for data augmentation. The main branch of the model is initialized with the model weights pre-trained on ImageNet^40^. The segmentation branch is randomly initialized and trained from scratch. Both branches are trained using the same street view image dataset with image-level binary labels as supervisory signals.

To estimate the directions of power lines in an image, we apply Hough transform^41^ on the CAMs generated by the line detector. Hough transform can extract lines and estimate their directions in a CAM. In order to detect multiple lines in an image, once a line is detected, we remove it from the CAM by adding a mask and re-apply Hough transform to the CAM, until all lines in the image have been detected. Similarly, for estimating pole orientations, we also apply Hough transform to the CAM generated by the pole detector and calculate the angle between the detected pole and the horizontal axis of the image (see Supplementary Fig. 3).

To predict exact geo-coordinates of poles, we assume utility poles are approximately perpendicular to the ground, hence any pole in an upward street view must point to the image center. Under this assumption, the angle between the detected pole and the horizontal axis of the north-facing image represents the pole orientation. By drawing multiple rays of pole orientations starting from street view points and intersecting these rays, the exact locations of poles can be derived (Fig. 2b). Intersecting two rays can obtain a single intersection point, while intersecting three or more rays can potentially obtain multiple intersections and we use spatial clustering to merge intersections that are close to each other.

### Road modeling and link prediction

We further integrate road information to enrich the features for predicting whether there is a line connection between two predicted poles. Specifically, each road can be represented as a series of line segments. If a detected pole or a street view point has a distance ≤*D*_attach_ to a road, it will be attached to that road (Fig. 2c). All attached street view points and poles are sorted in order along the road. Moreover, to reduce the number of poles missed by the pole detector, we insert pole(s) between a pair of poles if the distance between them is greater than a threshold *D*_insert_.

We develop a link prediction model that takes feature variables for a pair of poles as inputs and outputs whether there is a line connection between the pole pair. Any pair of poles with a distance less than a threshold *D*_cand_ is considered as a candidate. We consider various types of classification models including logistic regression, decision tree, random forest, support vector machine, and gradient boosting. Feature candidates include (1) Distance between the two poles. (2) Whether the two poles are on the same road. (3) Whether the two poles are next to each other along the road. (4) Fraction of street view images with power lines detected between the two poles. (5) Minimum and average differences between the power line directions estimated from street view images and the direction of the line connecting the two poles. The small difference gives evidence that there is a power line connecting the two poles. (6) Whether either of the poles is detected by the pole detector or inserted. (7) Whether either of the poles is at a road intersection. (8) Whether the two poles are at the same road intersection. (9) The binary prediction of the modified Dijkstra’s algorithm^8,26^ running on a raster map. This algorithm finds the most efficient paths to connect poles: On a geospatial raster, each cell is assigned with a weight. By setting the weights at the positions of roads to be lower than others, connecting poles along a road is preferable (see the weight assignment scheme in Supplementary Note 1).

The best feature set and hyperparameters for link prediction are selected based on the 9-fold cross-validation on the San Carlos development set which are divided into 9 subsets according to the boundary divisions of the 9 census tracts in San Carlos. The output of the link prediction module is a geospatial graph with predicted poles as nodes and predicted line connections as edges.

### Underground grid mapping

Street view images are only able to capture the information of overhead distribution grids. To predict the grid map for areas where power lines are underground or street view images are not available, we develop a heuristic approach that integrates the information of the predicted overhead grid map, road networks, and building locations. An assumption for this approach is that all buildings should be connected to grids, which means that buildings that are not connected to overhead grids should be connected by underground grids. Therefore, this approach is only applicable to regions with 100% electricity access. Under this assumption, we predict the underground grid map by first identifying buildings which cannot be reached by the predicted overhead grid, and then running a modified Dijkstra’s algorithm^8,26,27^ to generate paths to greedily connect them. The paths generated in this algorithm are used as the prediction of the underground grid.

To pick out unconnected buildings, we dilate the line connections of the predicted overhead grid with a radius *R*_dilate_ (*R*_dilate_ = 70m) and overlay it with the building map (Fig. 2d). Here we assume a building is connected to the grid through its nearby power lines, so that buildings that are not covered by the dilated paths cannot be connected to the predicted overhead grids within the distance *R*_dilate_. In the modified Dijkstra’s algorithm, these unconnected buildings are set as the targets to be connected by underground grids, and new paths are greedily generated on top of the predicted overhead grid until all targets are connected. The algorithm is run on a raster map where the predicted overhead grid, roads, and buildings are all discretized. Each cell in the geospatial raster is assigned with a weight. Paths can be generated from one cell to any of its 8 neighbor cells (including diagonal neighbors). The objective of the algorithm is to find the paths to connect all targets with the minimum total weight. By setting the weights of road cells to be lower than those of other cells, connections following roads are preferable (see the weight assignment scheme in Supplementary Note 1). Such a weight assignment is based on the grid construction practice that underground power lines are usually buried along roads to facilitate maintenance^42^. The final output of underground grid mapping is a 2D array with binary values indicating whether a cell belongs to the underground grid or not.

Such a heuristic grid mapping method can also be used to predict the map of distribution grids which do not have nearby street view images. To this end, other geospatial information besides the road map, such as topographic maps and land cover maps, can be integrated as prior knowledge to design the weight assignment scheme in the modified Dijkstra’s algorithm.

### Evaluation metrics

To compare a set of ground truth geolocations of poles *P* = {*p*_1_, *p*_2_, . . . , *p*_*M*_} with a set of predicted geolocations of poles *Q* = {*q*_1_, *q*_2_, . . . , *q*_*N*_}, we match all possible pairs of poles from the two sets ${\{({p}_{i},{q}_{j})\}}_{1\le i\le M,1\le j\le N}$ and sort them in ascending order of their pairwise geospatial distances. Given a distance threshold *D*_matching_, we pick pole pairs out of the sorted list starting from the first pair with the minimum distance, and add them to the list of matched pairs until the pairwise distance becomes greater than *D*_matching_. If either the predicted pole *q*_*j*_ or the ground truth pole *p*_*i*_ in a pair has already been picked before, this pair will be dropped and not picked again to avoid repetition. This can be viewed as a Bipartite Matching problem with greedy matching of pole pairs with the smallest distance. Then we use precision and recall to measure the pole localization performance, defined as:

1 $$\,{{\mbox{precision of pole localization}}}\,=\frac{\#\,\,{{\mbox{matched pairs}}}\,}{N}$$

2 $$\,{{\mbox{recall of pole localization}}}\,=\frac{\#\,\,{{\mbox{matched pairs}}}\,}{M}$$

To evaluate the link prediction performance of overhead grids, we compare the ground truth edge set *E* with the edge set *F* predicted by the link prediction model. Specifically, we define the precision and recall for link prediction as:

3 $$\,{{\mbox{precision of link prediction}}}\,=\frac{|E\cap F|}{|F|}$$

4 $$\,{{\mbox{recall of link prediction}}}\,=\frac{|E\cap F|}{|E|}$$

Here ∣∣ means the number of elements in a set. Note that edges between false negative poles (poles that are not detected) are counted as false negative edges, and edges between false positive poles (wrongly-detected poles) are counted as false positive edges. Moreover, false negative or false positive poles between two true positive poles along the same power line do not affect the overall grid topology. For example, a predicted power line connection *q*_*m*_ − *q*_*n*_ can be viewed as a correct prediction of ground truth connections *p*_*i*_ − *p*_*j*_ − *p*_*k*_ if *p*_*i*_ matches *q*_*m*_ and *p*_*k*_ matches *q*_*n*_. Similarly, predicted power line connections *q*_*l*_ − *q*_*m*_ − *q*_*n*_ can also be viewed as a correct prediction of a ground truth connection *p*_*i*_ − *p*_*j*_ in *E* if *p*_*i*_ matches *q*_*l*_ and *p*_*j*_ matches *q*_*n*_. To give tolerance to such mismatches, we measure the precision and recall after matching the equivalent segments from *E* and *F*.

We evaluate the overall grid mapping performance on a raster map since the underground part of the grid cannot be explicitly represented as nodes and edges. To this end, both the ground truth map and the predicted grid map—including overhead and underground parts—are discretized into 2D binary arrays with the cell size 2m × 2m, denoted as *G* and *H*, respectively. Cells belonging to grids have value 1 and otherwise 0. To calculate the correct rate of the predicted grid map (“precision”), we dilate the 1-value cells in *G* with a radius *R*_eval_ to generate *G*_dilate_, then overlay *G*_dilate_ with *H*, and calculate the ratio of 1-value cells in *H* that can be covered by the 1-value cells in *G*_dilate_. Similarly, to calculate the ratio of the ground truth grid map that can be detected within a distance (“recall”), we dilate the 1-value cells in *H* with the same radius *R*_eval_ to generate *H*_dilate_, then overlay *H*_dilate_ with *G*, and calculate the ratio of 1-value cells in *G* that can be covered by the 1-value cells in *H*_dilate_. Hence the precision and recall for overall grid mapping can be defined as:

5 $$\,{{\mbox{precision of grid mapping}}}=\frac{|{G}_{{{\mbox{dilate}}}}\cap H|}{|H|}$$

6 $$\,{{\mbox{recall of grid mapping}}}=\frac{|G\cap {H}_{{{\mbox{dilate}}}}|}{|G|}$$

Here ∩ means the intersection between two 2D binary arrays, and ∣∣ means the number of 1-value cells in a binary array.

### Supplementary information

Supplementary Information

### Source data

Source Data

## Data Availability

The data utilized or generated in this study have been deposited in 10.6084/m9.figshare.22723171. They can also be downloaded following the README file of the code repository https://github.com/wangzhecheng/GridMapping. The links to the original data sources for grid, roads, and buildings have been cited as references. Street view images in the test areas can be retrieved by running the code in the code repository. Source data are provided with this paper.

## Code availability

The source code is available at https://github.com/wangzhecheng/GridMapping.

## References

[1] International Energy Agency. SDG7: Data and Projections. https://www.iea.org/reports/sdg7-data-and-projections (Accessed: 2021-05-04).

[2] Farquharson D, Jaramillo P, Samaras C (2018). Sustainability implications of electricity outages in sub-Saharan Africa. Nat. Sustain..

[3] World Bank Group. Enterprise surveys. http://www.enterprisesurveys.org (Accessed: 2021-05-05).

[4] Frost & Sullivan. Growth opportunities in distributed energy, forecast to 2030. https://www.reportlinker.com/p05894509/?utm_source=GNW (Accessed: 2021-05-05).

[5] U.S. Energy Information Administration. U.S. energy mapping system. https://www.eia.gov/state/maps.php (Accessed: 2021-05-05).

[6] Liao Y, Weng Y, Liu G, Rajagopal R (2018). Urban MV and LV distribution grid topology estimation via group lasso. IEEE Trans. Power Appar. Syst..

[7] Gurara, D., Klyuev, V., Mwase, N. & Presbitero, A. Trends and challenges in infrastructure investment in developing countries. *International Development Policy∣ Revue Internationale De Politique De Dévelopement*. (2018).

[8] Arderne C, Zorn C, Nicolas C, Koks E (2020). Predictive mapping of the global power system using open data. Sci. Data.

[9] Haklay M, Weber P (2008). Openstreetmap: User-generated street maps. IEEE Pervasive Comput..

[10] Deka D, Backhaus S, Chertkov M (2017). Structure learning in power distribution networks. IEEE Transa. Control. Netw. Syst..

[11] Deka, D., Backhaus, S. & Chertkov, M. Estimating distribution grid topologies: A graphical learning based approach. *2016 Power Systems Computation Conference (PSCC)*. pp. 1–7 (2016).

[12] Weng Y, Liao Y, Rajagopal R (2016). Distributed energy resources topology identification via graphical modeling. IEEE Trans. Power Syst..

[13] Yu J, Weng Y, Rajagopal R (2017). PaToPa: A data-driven parameter and topology joint estimation framework in distribution grids. IEEE Trans. Power Syst..

[14] Yu J, Weng Y, Rajagopal R (2018). PaToPaEM: A data-driven parameter and topology joint estimation framework for time-varying system in distribution grids. IEEE Trans. Power Syst..

[15] Scully, P. Smart Meter Market 2019: Global penetration reached 14%-North America, Europe ahead. https://iot-analytics.com/smart-meter-market-2019-global-penetration-reached-14-percent (2019).

[16] LeCun Y, Bengio Y, Hinton G (2015). Deep learning. Nature.

[17] Elvidge C, Baugh K, Zhizhin M, Hsu F, Ghosh T (2017). VIIRS night-time lights. Int. J. Remote Sens..

[18] Schmidt E, Bhaduri B, Nagle N, Ralston B (2018). Supervised classification of electric power transmission line nominal voltage from high-resolution aerial imagery. GISci. Remote Sens..

[19] Gomes M (2020). Mapping utility poles in aerial orthoimages using atss deep learning method. Sensors.

[20] Huang B (2021). GridTracer: Automatic mapping of power grids using deep learning and overhead imagery. IEEE J. Sel..

[21] Zhang W (2018). Using deep learning to identify utility poles with crossarms and estimate their locations from google street view images. Sensors.

[22] Krylov V, Kenny E, Dahyot R (2018). Automatic discovery and geotagging of objects from street view imagery. Remote Sensing.

[23] Kim J, Kamari M, Lee S, Ham Y (2021). Large-scale visual data-driven probabilistic risk assessment of utility poles regarding the vulnerability of power distribution infrastructure systems. J. Constr. Eng. Manag..

[24] Tang, Q., Wang, Z., Majumdar, A. & Rajagopal, R. Fine-grained distribution grid mapping using street view imagery. *NeurIPS 2019 Workshop on Tackling Climate Change With Machine Learning*. https://www.climatechange.ai/papers/neurips2019/31 (2019).

[25] Nesbit, J. The guide to off-grid homes. https://realestate.usnews.com/real-estate/articles/the-guide-to-off-grid-homes (Accessed: 2022-12-17).

[26] Gershenson, D., Rohrer, B. & Anna, L. A new predictive model for more accurate electrical grid mapping. https://code.fb.com/connectivity/electrical-grid-mapping (Accessed: 2020-03-01).

[27] Dijkstra, E. A note on two problems in connexion with graphs. *Edsger Wybe Dijkstra: His Life, Work, and Legacy*. pp. 287–290 (2022).

[28] Pacific Gas and Electric Company. Distributed Resource Planning (DRP) data and maps. https://www.pge.com/en_US/for-our-business-partners/distribution-resource-planning/distribution-resource-planning-data-portal.page (Accessed: 2020-02-19).

[29] The World Bank. World Bank country and lending groups. https://datahelpdesk.worldbank.org/knowledgebase/articles/906519-world-bank-country-and-lending-groups (Accessed: 2023-06-14).

[30] CAL FIRE 2021. Incident archive. https://www.fire.ca.gov/incidents/2021 (Accessed: 2021-04-27).

[31] Cordova, G. Cal Fire investigators point to tree-hitting PG&E power lines as cause of Dixie Fire. https://www.abc10.com/article/news/local/wildfire/dixie-fire-cause-pacific-gas-and-electric/103-03d568e1-b141-48a1-9579-713688a71826 (Accessed: 2022-05-01).

[32] Hall, K. Out of sight, out of mind: an updated study on the undergrounding of overhead power lines. *Edison Electric Institute, Washington, DC*. (2012).

[33] Unites States Census Bureau. Glossary. https://www.census.gov/programs-surveys/geography/about/glossary.html (Accessed: 2022-05-01).

[34] Neuhold, G., Ollmann, T., Rota Bulo, S. & Kontschieder, P. The mapillary vistas dataset for semantic understanding of street scenes. *Proceedings of The IEEE International Conference on Computer Vision*. pp. 4990–4999 (2017).

[35] Google Maps. Street View Static API. https://developers.google.com/maps/documentation/streetview (Accessed: 2022-12-17).

[36] World Bank Group. Africa - Electricity transmission and distribution grid map. https://datacatalog.worldbank.org/search/dataset/0040465 (Accessed: 2020-09-01).

[37] Microsoft Maps. Microsoft open building footprints dataset. https://github.com/microsoft/USBuildingFootprints (Accessed: 2022-02-01).

[38] Szegedy, C., Vanhoucke, V., Ioffe, S., Shlens, J. & Wojna, Z. Rethinking the Inception architecture for computer vision. *Proceedings of the IEEE Conference on Computer Vision and Pattern Recognition*. pp. 2818–2826 (2016).

[39] Zhou, B., Khosla, A., Lapedriza, A., Oliva, A. & Torralba, A. Learning deep features for discriminative localization. *Proceedings of the IEEE Conference on Computer Vision and Pattern Recognition*. pp. 2921–2929 (2016).

[40] Deng, J. et al. Imagenet: A large-scale hierarchical image database. *2009 IEEE Conference on Computer Vision and Pattern Recognition*. pp. 248-255 (2009).

[41] Illingworth J, Kittler J (1988). A survey of the Hough transform. Comput. Graph. Image Process..

[42] McCarthy, K. Undergrounding electric lines. *OLR Research Report*. pp. 2011-R-0338 https://www.cga.ct.gov/2011/rpt/2011-R-0338.htm (2011).

