# Supplementary material for: Geospatial mapping of distribution grid with machine learning and publicly-accessible multi-modal data
Source: Nat Commun. 2023 Aug 17;14:5006. doi: 10.1038/s41467-023-39647-3 (PMC10435496; doi:10.1038/s41467-023-39647-3)
Supplement: Supplementary file 1 — Supplementary Information [file 41467_2023_39647_MOESM1_ESM.pdf]

Geospatial Mapping of Distribution Grid with Machine Learning and Publicly-Accessible Multi-Modal Data

Supplementary Information

Zhecheng Wang,[1,2] Arun Majumdar,[3,4,*] and Ram Rajagopal[1,2,*]

[1]Department of Civil & Environmental Engineering, Stanford University, Stanford, CA 94305, USA

[2]Department of Electrical Engineering, Stanford University, Stanford, CA 94305, USA

[3]Department of Mechanical Engineering, Stanford University, Stanford, CA 94305, USA

[4]Department of Energy Science & Engineering, Stanford University, Stanford, CA, 94305, USA

*E-mail: amajumdar@stanford.edu; ramr@stanford.edu

Supplementary Note 1. Weight assignments for the modified Dijkstra's algorithm

The modified Dijkstra's algorithm predicts power line connections by finding the paths with the minimum total weight to connect poles (for overhead grid) or buildings (for underground grid). Note that for overhead grids, the output paths generated by this algorithm are not directly used as the predictions of power line connections. Instead, they are used as a feature input of the link prediction model (see Methods in the main paper).

We discretize both targets (poles or buildings) and roads onto a raster map. On this geospatial raster, each cell is assigned with a weight. A lower weight of a cell indicates higher preference for using this cell in a path to connect the targets. The weight assignments for different types of roads specified by the "highway" class in OpenStreetMap are: motorway: 1/10, motorway link: 1/10, primary: 1/8, primary link: 1/8, secondary: 1/7, secondary link: 1/7, tertiary: 1/6, tertiary link: 1/6, unclassified: 1/5, residential: 1/4, living street: 1/4, cycleway: 1/4, service: 1/4, footway: 1/3, track: 1/3, pedestrian: 1/3, path: 1/2, highways not belonging to any of the above types: 2/3. Non-highway: 1.

Such weight assignments are based on the intuition that power lines are more likely to follow a more important road (e.g., primary) than a less important one (e.g., footway) hence the weight assigned to the former is lower than the latter. We also tune and try different sets of weight assignments on the development set but the performance difference is small.

In this work, we only use road networks as a prior knowledge to design the weight assignment scheme. Other geospatial information, such as topographic maps and land cover maps can also be integrated into weight assignments to capture the grid construction preference, which deserves future exploration.

Supplementary Note 2. Sources of error and potential approaches for mitigation

Two types of errors occur in the grid mapping: (1) false positive (FP) mapping of utility poles and power line connections which does not match the ground truth distribution grid, and (2) utility poles and power

line connections missed by the model (false negative (FN) mapping). The statistics of sources of error for the California test areas and SSA test areas are shown in Supplementary Figure 4 and Supplementary Figure 5, respectively.

For the California test areas (Supplementary Figure 4), over 80% of the FP errors and over 70% of the FN errors come from the underground grid mapping, which is not surprising due to the heuristic nature of the underground grid mapping approach. A potential mitigation approach is to incorporate other geospatial information besides the road network into the weight assignment of the modified Dijkstra's algorithm. For example, topological and land cover maps can be included to provide more information on the difficulty of undergrounding construction on different parts of the land, which deserves future exploration. For the overhead part, 55% of the FP errors and 73% of the FN errors come from pole detection. Although pole insertion can reduce the number of poles missed by the pole detector, it contributes to 47% of the FP pole detection. Other significant sources of FP errors in pole detection include the poles detected in a close distance to a true positive detected pole which are deemed as duplication (26%), and false positive classification of street view images made by the CNN model (18%). Common FP samples that confuse the CNN model include street light poles and telegraph poles without power lines attached, which can be potentially mitigated by including more hard negative samples of these confusing objects into the training set.

For FN errors in pole detection (Supplementary Figure 4), poles that cannot be captured by the street view images (35%) and missing detection of poles in images are the major sources of error (52%). A potential approach to reduce such errors is to use upward street view images with a broader field of view (FoV) to capture poles that are far away from street view points. The FoV currently used in this work is 120º but broader FoV can be obtained by processing the panoramic street view images which are commonly captured in street view photography.

Compared to the California test areas, a larger fraction of FN errors comes from pole detection (84% compared to 73%) in the SSA test areas (Supplementary Figure 5), and a larger fraction of the FN pole detections can be attributed to the incorrect prediction of CNN model on street view images (71% compared to 52%). One reason is that utility poles in SSA can have different visual features from those in the U.S. where the model is trained. Collecting and including pole samples in SSA for model fine-tuning can potentially mitigate such errors. Few-shot learning can be used to reduce the required number of training images for SSA. Another reason is that the heights of utility poles are generally shorter in SSA than those in the U.S. which make them more difficult to identify in street view images. This can also be alleviated by using street view images with broader FoV.

Supplementary Note 3. Street view images

Street view images used in this research are retrieved with Google Street View Static API[1]. Given a pair of latitude and longitude, we first use the API to request the metadata to check whether a street view image is available at this given location. If available, we then use the API to retrieve the upward street view image (set pitch = 90º) at that location. When specifying the parameters of API queries, the field of view (FoV) is set to be 120º, the heading is set to be 0º (i.e., facing north), and the image size is set to be 640 × 640 pixels.

Each image has three bands (RGB). Alternative street view image sources include Mapillary[2], Microsoft Bing Maps Imagery API[3], and Baidu Maps Static API[4] with similar parameter specifications.

For training and test sets, the locations of images are randomly sampled from the San Francisco Bay Area. For model deployment, each test area is discretized as a 10m × 10m mesh so that image locations are sampled every 10m from north to south and every 10m from east to west. We use the API to check the availability of the street view image at each sampled location and retrieve the image if it is available. In Supplementary Note 5, we discuss a potential approach to reduce the number of images needing to be sampled.

Supplementary Note 4. Model evaluation in more countries

To further evaluate the generalizability of the proposed framework in different countries, we construct another image-level test set containing test areas in six countries (South Africa, Ghana, Vietnam, India, Brazil, Mexico City) from Africa, Asia, and Latin America. Each test area contains 300 street view images with randomly-sampled geo-locations. Each image is manually labeled with the presence/absence of pole/lines. Here we only test the model at the image level rather than the grid system level as the ground-truth distribution grid maps are currently not available for these test areas. The performance is shown in Supplementary Table 2. We find that although the model has not been trained with images in any of these cities, it can still maintain a good performance (F1 score > 80%) when being transferred to these areas. Note that the performance is moderately lower in the test area in Bengaluru, India due to more entanglement of lines, which can be improved by fine-tuning the model with local samples.

Supplementary Note 5. A potential approach to reduce the number of images needed for pole localization

Pole placement usually follows a certain pattern, which can be incorporated into the model as prior knowledge for reducing the number of images needed to be sampled to localize poles. Supplementary Figure 7 shows the distribution of the distances between adjacent poles for the California test areas, where we can find that most poles are spaced 30-60m apart. Furthermore, the variance of pole distances is even smaller along the same road. Supplementary Figure 8 shows the geospatial map of the pole placement along different roads in the test area of Salinas, California, from which we can observe that the distances of poles along the same road have comparatively small variance. Therefore, a potential approach to reduce the number of images needed can follow this procedure: First, sample a few street view images along a road by applying large sampling intervals (i.e., larger than 10m as used in the current framework) to identify the part of the road that has power lines. Second, choose a small fraction of the road where power lines are detected, and sample images at a dense interval (e.g., 10m) to detect utility poles and calculate their spacing distance. Third, use this spacing distance to predict the locations of other poles along this road. This approach has the potential to reduce the image acquisition burden and computation time (Supplementary Table 1), which deserves future exploration and validation.

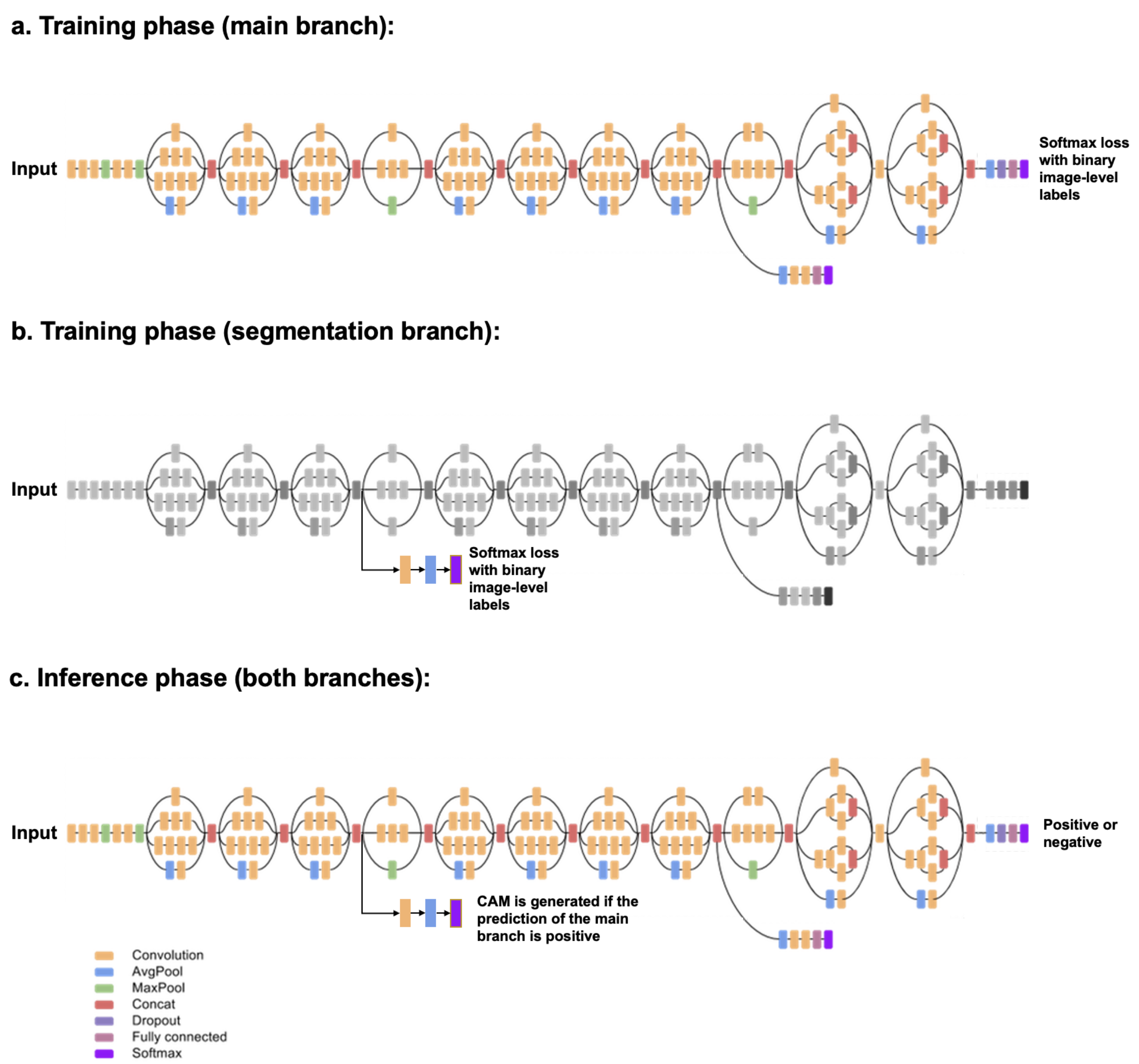

Supplementary Figure 1. The model architecture and training/inference phases. They are applied to both the line detector and the pole detector. The main branch is an Inception-v3 architecture with the output of binary class prediction. **a.** Training phase for the main branch. The loss function is the Softmax loss by comparing the prediction against the image-level binary labels. Adam optimizer is used for training. **b.** Training phase for the segmentation branch. After the main branch is trained, a segmentation branch (“convolutional layer-average pooling layer-Softmax” structure) is added to an intermediate layer of the main branch, and this segmentation branch is also trained with the Softmax loss by comparing the prediction against the image-level binary labels, while the main branch is kept frozen (denoted in grayscale). Adam optimizer is used for training. **c.** Inference (model deployment) phase. We use the output of the main branch to classify an image into either positive or negative. If it is positive, then the segmentation branch is executed to generate the Class Activation Map (CAM).

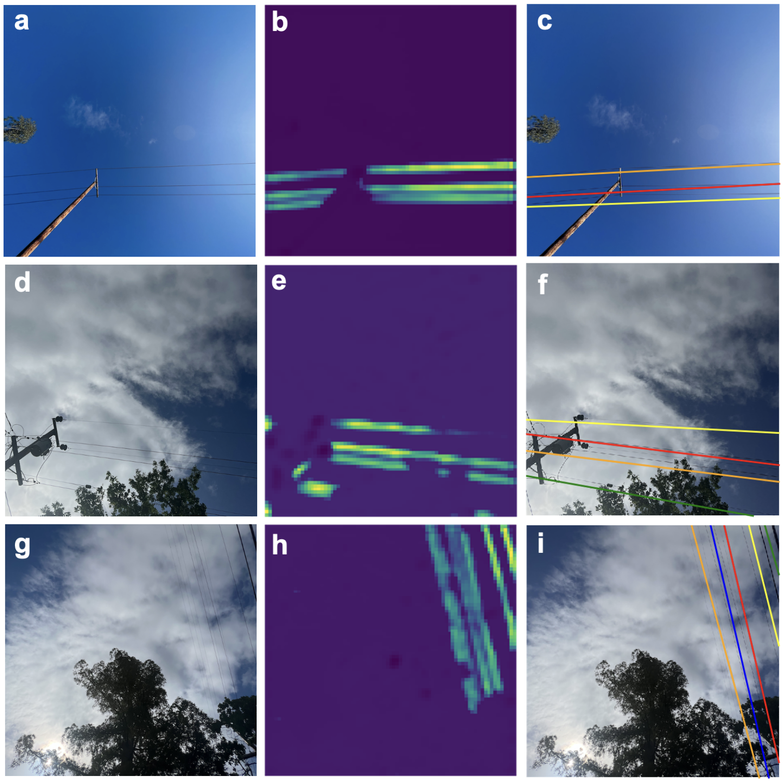

Supplementary Figure 2. Examples of power line detection. The left column (**a**, **d**, **g**) shows the input images. The middle column (**b**, **e**, **h**) shows their corresponding Class Activation Maps (CAMs) for power line extraction. The right column (**c**, **f**, **i**) shows the extracted line directions after Hough Transform.

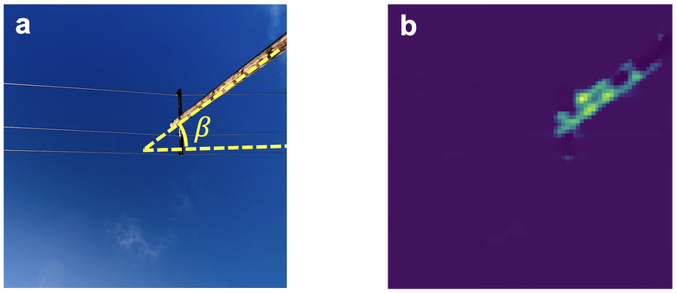

Supplementary Figure 3. An example of pole orientation estimation. a. An example image that contains a utility pole, where β represents the orientation of the pole. **b.** The corresponding Class Activation Map (CAM) for estimating the pole orientation.

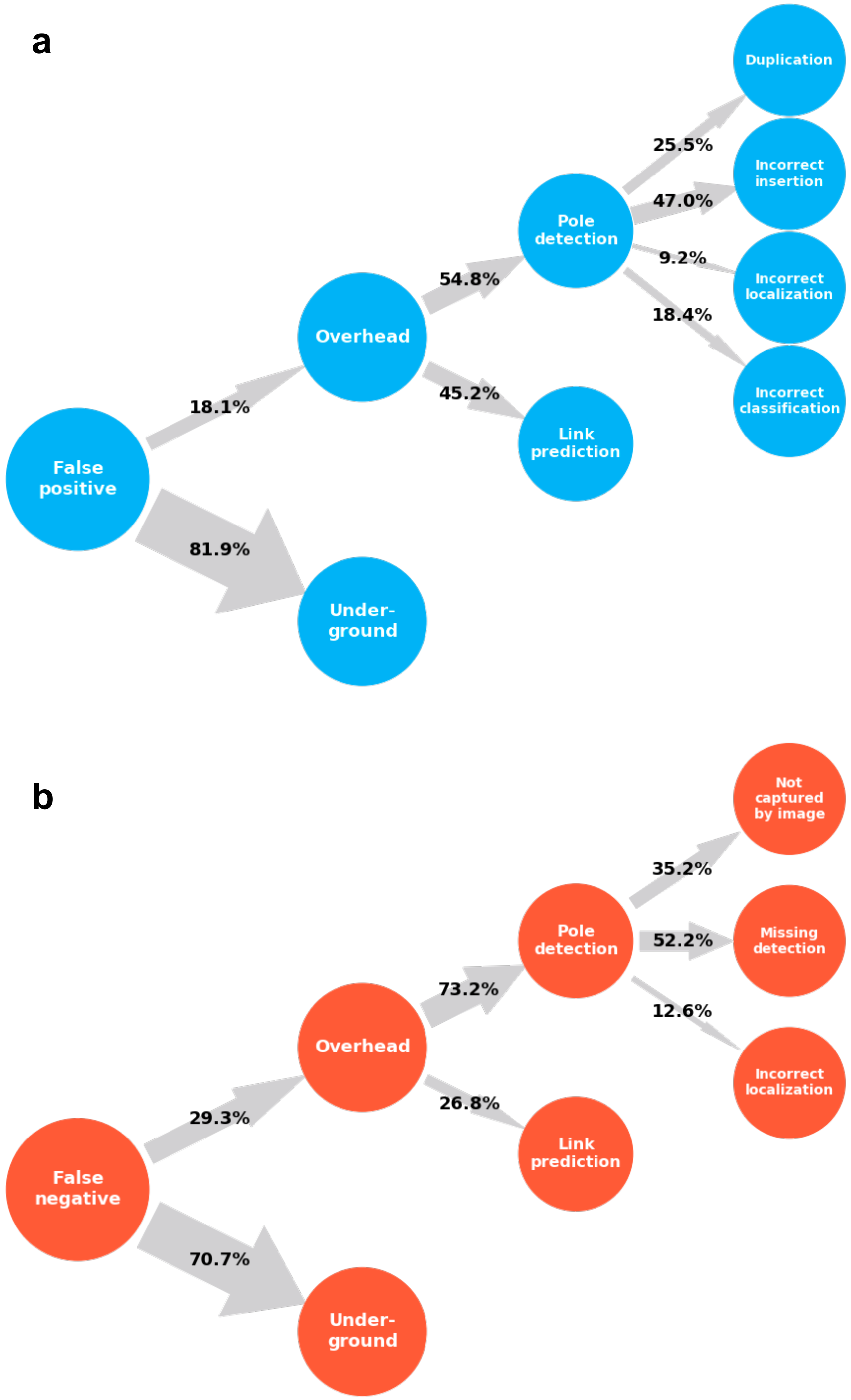

Supplementary Figure 4. Sources of error in overall grid mapping in the California test areas. Both overhead and underground parts are included. The results across the 5 test areas (Newark, Santa Cruz, Yuba City, Pacific Grove, and Salinas) are aggregated. **a.** False positive errors. **b.** False negative errors. “Duplication” means that a pole is detected in a close distance to a true positive detected pole so it is not counted as another true positive sample. “Incorrect classification” means the false positive errors made by the CNN model in identifying poles in street view images. “Missing detection” means the false negative errors made by the CNN model in identifying poles in street view images.

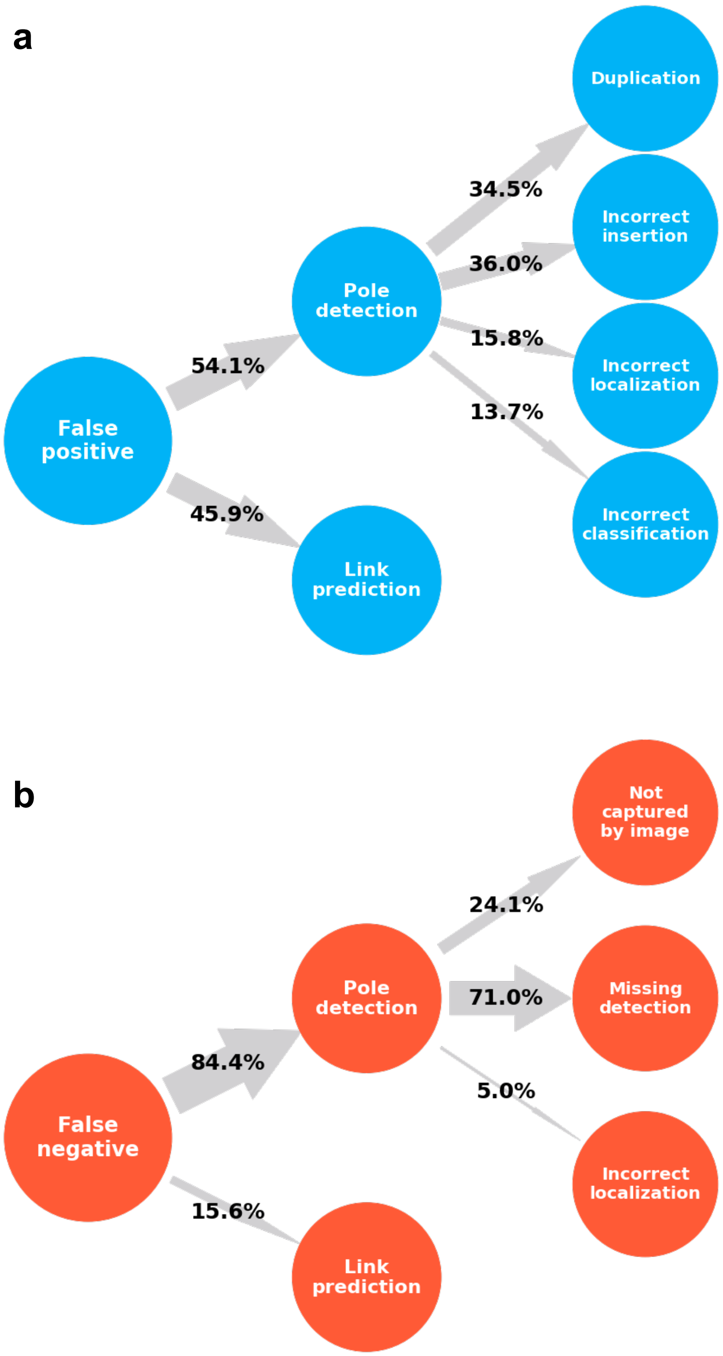

Supplementary Figure 5. Sources of error in overhead grid mapping in the Sub-Saharan Africa (SSA) test areas. The results across the 5 test areas are aggregated. **a.** False positive errors. **b.** False negative errors. "Duplication" means that a pole is detected in a close distance to a true positive detected pole so it is not counted as another true positive sample. "Incorrect classification" means the false positive errors made by the Convolutional Neural Network (CNN) model in identifying poles in street view images. "Missing detection" means the false negative errors made by the CNN model in identifying poles in street view images. Note that the sources of error are only available for overhead grid mapping in the SSA test areas, as underground grid mapping is not applied to the test areas in SSA.

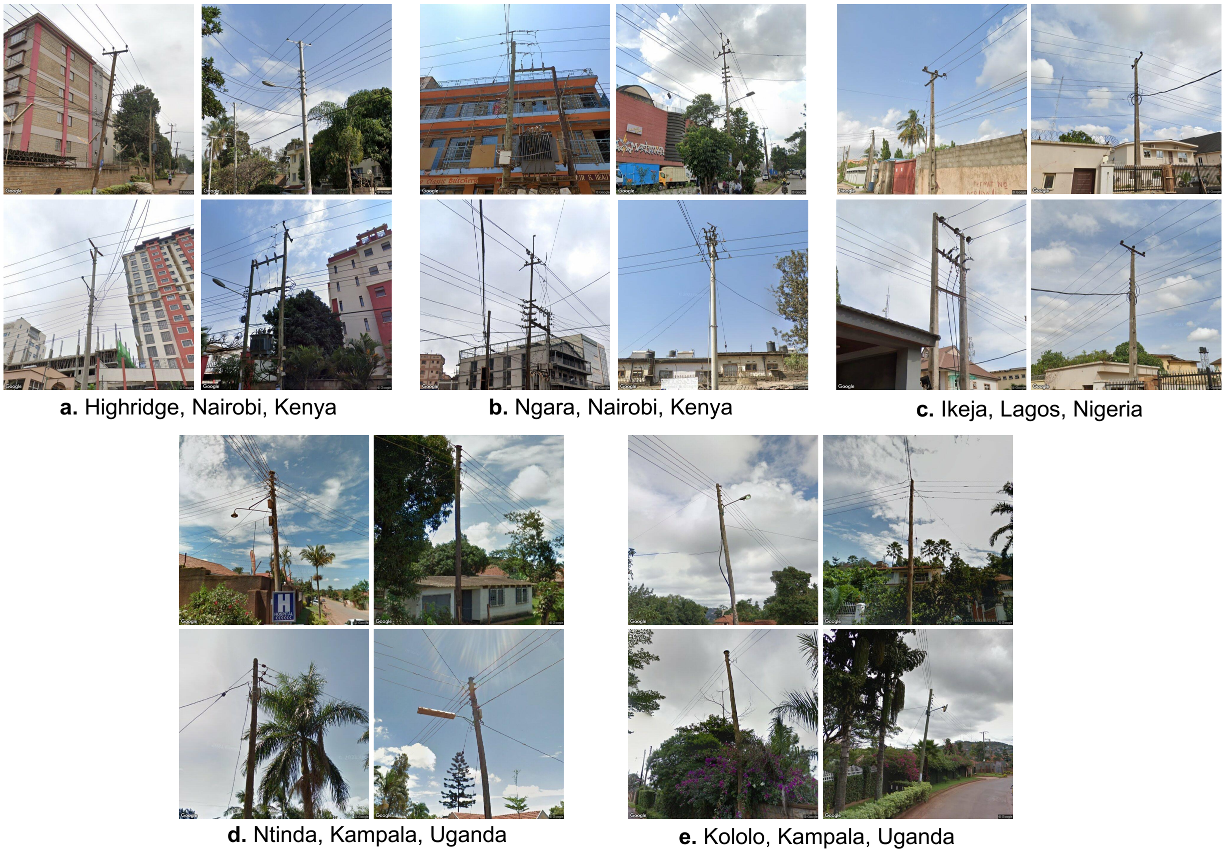

Supplementary Figure 6. Diverse types of utility poles covered in the Sub-Saharan Africa (SSA) test areas. Note that non-upward perspectives (pitch between 0º and 90º) are shown here for a better view of the visual appearance of different types of poles, while upward street views (pitch = 90º) are used by the model to reduce the amount of background objects (e.g., trees, buildings). These images are retrieved from Google Street View. **a.** Highridge, Nairobi, Kenya. **b.** Ngara, Nairobi, Kenya. **c.** Ikeja, Lagos, Nigeria. **d.** Ntinda, Kampala, Uganda. **e.** Kololo, Kampala, Uganda. The test areas in Kenya and Nigeria generally have taller utility poles with crossarms, which are usually present in urban areas or areas with relatively higher levels of economic development. By contrast, the test areas in Uganda generally have shorter and simpler utility poles without crossarms, which are commonly present in more rural areas or areas with lower levels of economic development.

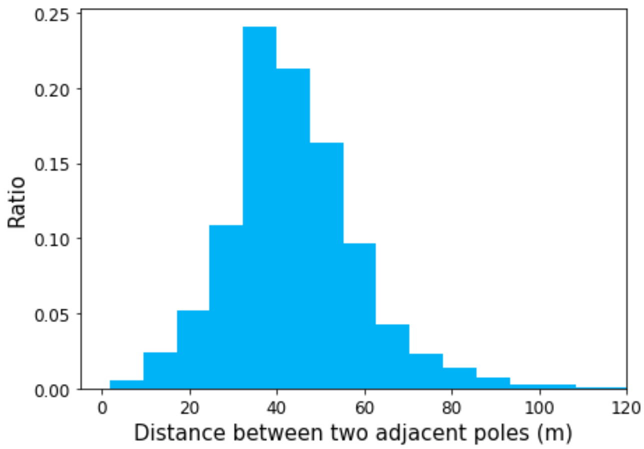

Supplementary Figure 7. The distribution of the distances between two adjacent poles for the California test areas. The mean value is 44.3m. The standard deviation is 16.6m. 10% and 90% percentiles are 26.8m and 62.4m, respectively.

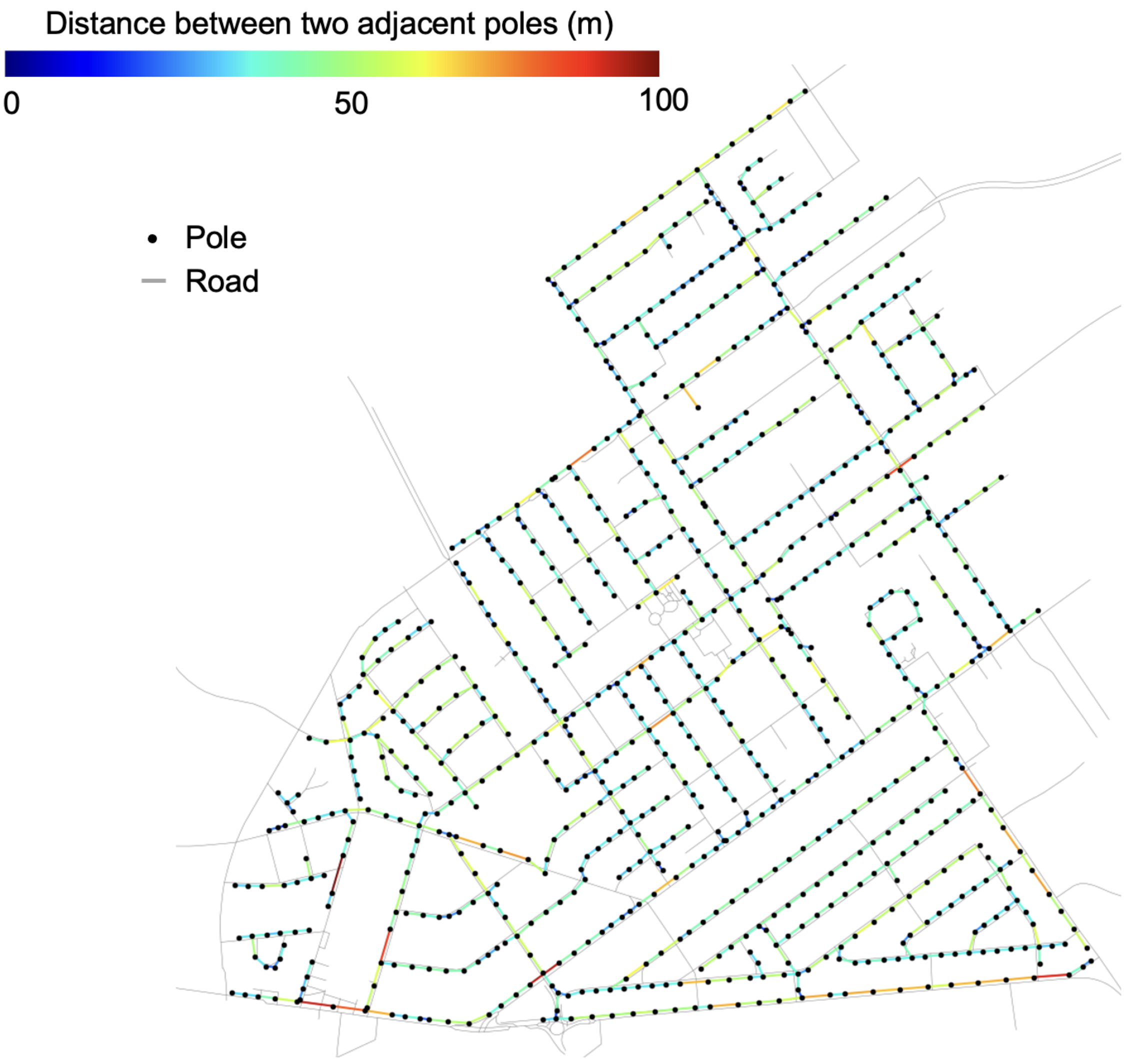

Supplementary Figure 8. The geospatial map of the pole placement along different roads in the test area of Salinas, California, U.S.A. Poles are represented as black dots. Roads are represented as gray line segments. Power lines are represented as line segments with different colors representing different distances between poles.

Test area	Total computation time (second)	Total power line length (km)	Computation time per unit of power line (second/km)
**a.** Test areas in Northern California			
Newark, CA, U.S.A.	1711	161	10.6
Santa Cruz, CA, U.S.A.	1286	107	12.0
Yuba City, CA, U.S.A.	1158	96	12.1
Pacific Grove, CA, U.S.A.	1435	75	19.1
Salinas, CA, U.S.A.	723	35	20.7
**b.** Test areas in Sub-Saharan Africa			
Ntinda, Kampala, Uganda	290	18	16.1
Kololo, Kampala, Uganda	462	33	14.0
Highridge, Nairobi, Kenya	292	33	8.8
Ngara, Nairobi, Kenya	266	16	16.6
Ikeja, Lagos, Nigeria	242	20	12.1

Supplementary Table 1. The overall computation time of grid mapping in each test area. The speed test is conducted on a single Intel Xeon Skylake CPU and a single NVIDIA Tesla V100 GPU. The computation time per km of power line ranges from 8.8 to 20.7 second/km.

Test area	Target	Precision	Recall	F1 score
Africa				
Cape Town, South Africa	Line	0.925	0.877	0.900
	Pole	0.911	0.932	0.921
Accra, Ghana	Line	0.948	0.928	0.938
	Pole	0.832	0.872	0.851
Asia				
Ho Chi Minh City, Vietnam	Line	0.856	0.941	0.896
	Pole	0.981	0.869	0.922
Bengaluru, India	Line	0.880	0.785	0.830
	Pole	0.842	0.826	0.834
Latin America				
São Paulo, Brazil	Line	0.953	0.961	0.957
	Pole	0.948	0.952	0.950
Mexico City, Mexico	Line	0.932	0.961	0.946
	Pole	0.813	0.942	0.873

Supplementary Table 2. Image-level model performance on additional test areas. For each test area, precision, recall, and their harmonic mean (F1 score) for both the line detector and the pole detector are reported. Both models can maintain a good performance (F1 score > 80%) in these test areas across three different continents of the world.

Supplementary References:

[1] Google Maps. Street View Static API. https://developers.google.com/maps/documentation/streetview
[2] Neuhold, G., Ollmann, T., Rota Bulo, S., & Kontschieder, P. (2017). The mapillary vistas dataset for semantic understanding of street scenes. In Proceedings of the IEEE International Conference on Computer Vision (pp. 4990-4999).
[3] Microsoft. Bing Maps Imagery API. https://learn.microsoft.com/en-us/bingmaps/rest-services/imagery
[4] Baidu Maps. Baidu Maps Static Panorama API. https://lbsyun.baidu.com/index.php?title=viewstatic
